# Ataxia with oculomotor apraxia type 2 due to the new compound heterozygous variants c.5825T>C (inherited) and c.6106+1G>T (de novo) in SETX

**DOI:** 10.1016/j.clinsp.2026.101146

**Published:** 2026-09-09

**Authors:** Ana C. Fiorini, Carla A. Scorza, Fulvio A. Scorza, Josef Finsterer

**Affiliations:** a Department of Speech Therapy, Escola Paulista de Medicina, Universidade Federal de São Paulo (EPM/UNIFESP), São Paulo, SP, Brazil; b Postgraduate Studies Program in Speech Therapy, Pontifícia Universidade Católica de São Paulo (PUC-SP), São Paulo, SP, Brazil; c Escola Paulista de Medicina, Universidade Federal de São Paulo (UNIFESP/EPM), São Paulo, SP, Brazil; d Neurology & Neurophysiology Center, Vienna, Austria

**Keywords:** Oculomotor apraxia, Spinocerebellar ataxia, Axonal neuropathy, SETX gene, Mutation

Dear Editor,

Ataxia with Oculomotor Apraxia type 2 (AOA2) is a slowly progressive, autosomal recessive disorder characterized by the triad of ataxia, oculomotor apraxia and sensorimotor neuropathy.^1^^,^^2^ Ocular apraxia manifests as an inability to perform rapid, voluntary horizontal eye movements (saccades), often forcing patients to make jerky head movements to change their gaze direction. Automatic (reflexive) eye movements, however, remain intact. AOA2 is caused by biallelic variants in the SETX, which encodes senataxin, a protein involved in DNA/RNA repair and essential for genomic stability.^1^^,^^2^ Mutations in the SETX gene lead to a reduction or complete absence of senataxin, depending on the mutation type.^1^^,^^2^ Neuropathy in AOA2 is usually axonal in nature.^3^^,^^4^ To date, only a few patients with AOA2 and axonal neuropathy have been described.^4^^,^^5^ A patient with AOA2 and axonal neuropathy due to the heterozygous variants c.5825T>C and c.6106+1G>T has not yet been published.

The patient is a 32-year-old man who, at the age of 28, was diagnosed with AOA2 based on the clinical presentation (oculomotor apraxia, double vision, ataxic posture and gait) and presence of the heterozygous mutations c.5825T>C (p.Ile1942Thr) (inherited from his mother) and c.6106+1G>T (de novo) in the SETX gene. The latter mutation was not detected in either the father or the mother. Nonsense-mediated genetic decay was expected. Testing for uniparental disomy was not performed. The patient’s medical history was also positive for arterial hypertension, diabetes, fatty liver disease, smoking and Sleep Apnoea Ayndrome (SAS). The mother denied any consanguinity with her husband. Biological paternity was confirmed by the mother's statement, however, molecular genetic confirmation (e.g., via microsatellite analysis) was not performed due to the father's unavailability. AOA2 first manifested at age 28, initially presenting with gait disturbances and headaches. From age 31, the patient also developed dysarthria with progressive speech impairment. Subsequently, he suffered from recurrent double vision, particularly when looking to the left. By age 30, he required a walking aid. At age 32, although he could eat and dress/undress independently, he required his mother's assistance with personal hygiene and toileting.

A clinical neurological examination performed by JF at age 32 revealed a sloping forehead, nystagmus when looking to the left, ocular apraxia when looking to the right or left, dysarthria, a quiet voice, neck muscle pain, slight past-pointing on the finger-to-nose test, dysdiadochokinesia, absent tendon reflexes, non-fixed flexion contractures of the fingers, mild leg ataxia, sensory disturbances (touch and vibration), ataxic stance with a non-directional tendency to fall, and a marked tendency to fall during the Unterberger stepping test. Blood tests showed elevated creatine kinase (323 U/L [n, < 190 U/L]), elevated transaminases, hypertriglyceridemia, severe vitamin D deficiency and an HbA1c level of 5.3. Alpha-fetoprotein was increased to 27 ng/mL (n, 0‒20 ng/mL). Video oculography was performed, but the raw data cannot be presented here. It showed spontaneous Downward Nystagmus (DBN) and nystagmus bilaterally on horizontal gaze. Additionally, macrosaccades and slowed vertical saccades were observed. Suppression of the Vestibulo-Ocular Reflex (VOR) was not demonstrable. Unfortunately, the video from the cinematography department was not made available. The right vertebral artery could not be visualized on carotid ultrasound. Nerve conduction studies showed severe axonal neuropathy (Table 1). Brain MRI showed mild supratentorial and infratentorial atrophy. At the time of examination, the patient was taking bisoprolol, esomeprazole and milk thistle extract.

**Table 1:** Results of nerve conduction studies at age 32.

	dL	NCV	Amplitude
Right tibial nerve	7.3ms	40m/s	0.08mV
Right peroneal nerve	5.5ms	36m/s	0.16mV
Left tibial nerve	5.1ms	37m/s	0.49mV
Left peroneal nerve	13.3ms	35m/s	0.11mV

dLL, Distal Latency.

The patient presented is interesting in several respects. Firstly, one of the compound heterozygous variants was novel. The variant c.5825T>C (p.Ile1942Thr), which he inherited from his mother, has already been reported,^6^ while the variant c.6106+1G>T is not recorded in any database. Besides the fact that this variant is a first occurrence, it could, although unlikely, also be due to germline mosaicism. The genotype-phenotype correlation of the variant c.5825T>C was straightforward as previously described. Second, the previously unreported variant c.6106+1G>T was not detected in either the father or the mother, suggesting spontaneous origin. Compound heterozygous variants, one of which was inherited and the other of which arised spontaneously, have so far only been reported in other diseases, but not in AOA2.^7^ The third point is that the patient also suffered from diabetes and SAS, which are common in the general population but have not previously been reported in association with SETX mutations. The fact that SETX variants are not only associated with AOA2, but are phenotypically heterogeneous and also manifest with familial ALS type 4,^8^ cerebellar ataxia with neuropathy and elevated alpha-fetoprotein,^9^ proximal spinal muscular atrophy,^10^ myoclonus and ataxia, and premature ovarian aging suggests that the mutation could be the cause of these features. However, diabetes and SAS are common in the general population and are unlikely to be causally related to the SETX mutation. Whether this is a true phenotypic extension of AOA2 or random comorbidities requires further observation. The phenotypic heterogeneity of SETX mutations poses a challenge for accurate diagnosis and genetic counseling.

In summary, the presented case expands the genotypic spectrum of AOA2 and should remind clinicians that both biallelic variants are not always inherited.

## Ethics statement

Not applicable.

## Data access statement

Not applicable.

## Compliance with ethics guidelines

This article is based on previously conducted studies and does not contain any new studies with human participants or animals performed by any of the authors.

## Authors’ contributions

JF: Design; Data generation; clinical examination; data collection; literature search; discussion; first draft; critical comments; final approval.

AF: Literature review; discussion; final approval.

FS: Literature review; discussion; final approval.

CS: Literature review; discussion; final approval.

## Funding

No funding was received.

## Declaration of competing interest

The author declares that the research was conducted in the absence of any commercial or financial relationships that could be construed as a potential conflict of interest.

## Data Availability

All data are available from the corresponding author.
